# Optimum stem length for mitigation of periprosthetic fracture risk following primary total knee arthroplasty: a finite element study

**DOI:** 10.1007/s00167-016-4367-8

**Published:** 2016-11-03

**Authors:** Noel Conlisk, Colin R. Howie, Pankaj Pankaj

**Affiliations:** 1 Orthopaedic Engineering, The University of Edinburgh, Edinburgh, UK; 2 School of Clinical Sciences, The University of Edinburgh, Edinburgh, UK; 3 School of Engineering, The University of Edinburgh, Edinburgh, UK; 4 Department of Orthopaedics, New Royal Infirmary of Edinburgh, Old Dalkeith Road, Little France, Edinburgh, UK; 5 Institute for Bioengineering, The University of Edinburgh, Faraday Building, The King’s Buildings, Edinburgh, EH9 3JL UK

**Keywords:** Optimum stem length, Distal femur, Total knee arthroplasty, Finite element, Periprosthetic stress

## Abstract

**Purpose:**

Due to age-related changes to the material properties and thinning of the cortical bone structure, older patients with osteoporosis may be at greater risk of femoral fracture following total knee arthroplasty. This study investigates whether there is a potential role for stemmed prostheses in such scenarios to help mitigate peri-implant fracture risk, and if so what should the optimum stem length be to balance surgical bone loss with reduced fracture risk.

**Methods:**

Finite element models of the distal femur implanted with four different implant types: a posterior stabilising implant, a total stabilising implant with short stem (12 mm × 50 mm), a TS implant with medium stem (12 mm × 75 mm), and a TS implant with long stem (12 mm × 100 mm), were developed and analysed in this study. Osteoporotic properties were applied to the implanted femurs and the periprosthetic stresses and strains of each were recorded.

**Results:**

All stem lengths examined were found to lead to a reduction in periprosthetic stress in comparison with a primary stemless implant, with short-, medium-, and long-stemmed implants leading to an 11, 26, and 29% reduction in stress, respectively.

**Conclusion:**

The results of this study show that periprosthetic stress and therefore fracture risk in old osteoporotic patients may be reduced through the use of stemmed femoral components. Of the three stems investigated, a medium-length stem is found to represent the best balance between bone preservation at the time of surgery and reduction in periprosthetic stress following implantation.

## Introduction

A rare but potentially devastating failure mode of primary total knee arthroplasty (TKA) is periprosthetic fracture. This mode of failure can arise due to a number of different factors such as: a direct trauma to the replaced joint resulting from a low-velocity fall or car accident [26], loss of supporting bone [29, 33] due to stress shielding, osteolysis and osteoporosis, and an increase in localised stress concentrations due to loosening [21]. Older patients with osteoporosis may be particularly at risk of femoral fracture following TKA, due to alterations in the material properties of the bone as a result of ageing, and thinning of the cortical bone structure through endosteal trabecularisation [6, 28]. The current incidence of periprosthetic fracture worldwide following primary TKA is believed to be in the range of 0.6–3.0% [21, 26, 32]. However, this failure mode has the potential of becoming a more serious clinical issue as the population ages and a greater proportion of younger more active patients undergo TKA.

Traditionally, stemmed femoral prostheses are used for revision of failed primary TKAs. In such scenarios, the stem helps to align the prosthesis, aid implant stability in the presence of bone loss [24] and protect bone grafts prior to integration with the host bone through load sharing at the interface [10]. However, the initial stability afforded by the use of large-diameter diaphyseal engaging stem configurations may lead to an increase in bone loss over time as a result of greater levels of stress shielding distally [9, 38]. Studies on cemented non-diaphyseal engaging stems report a slightly more favourable outcome in terms of the level of reported stress shielding [9, 38], in comparison with large canal filling stems.

Careful consideration of the impact stemmed prosthesis can have on the mechanical environment of the surrounding bone is essential to a successful patient outcome when treatment requires stemmed prosthesis. By understanding the potential limitations of stems and their impact on the host environment, surgeons and engineers can leverage these devices to generate better clinical outcomes. In a recent in vitro study by Completo et al. [7], the authors suggested the use of a long press-fit stem as a means of reducing strain and therefore fracture risk at the notch edge following notching of the anterior femoral cortex during TKA, thus turning a normal disadvantage of stemmed prosthesis into a clear advantage for a particular clinically encountered scenario.

The present study investigates the application of femoral prostheses with cemented non-diaphyseal engaging stems to determine what influence stemmed implants have on the levels of stress in the region immediately above the implant, for a simulated old patient with osteoporosis, in comparison with primary stemless implants for the same patient. A secondary goal was to determine whether there was an optimum stem length to balance bone loss at the time of surgery with peri-implant stress reduction.

The main hypothesis of the study is that stemmed implants used in a primary setting may be beneficial in reducing periprosthetic stress and therefore mitigating some of the fracture risk associated with ageing and osteoporosis in older patients.

## Materials and methods

### Geometry

The femoral geometry used in this study was a three-dimensional virtual reconstruction [3] of the large left fourth-generation composite femur (Sawbones; Pacific Research Laboratories, Vashon, Washington, USA). This geometry was subsequently modified to accept a posterior stabilising (PS) implant, and a total stabilising implant (TS) with femoral stems of varying length. All implants were from the Triathlon^®^ series product line (Stryker^®^, Newbury, United Kingdom). Physical implant measurements and surgical theatre templates were used in conjunction with computer-aided design software (Autodesk Inventor 2010, Autodesk Inc. San Rafael, California, US) to develop 3D models of the femoral implant; the same software was also used to incorporate surgical cuts into the femur to accommodate each implant.

In this study, three different stem lengths were considered for the TS implanted femurs: a short stem (12 mm × 50 mm), a medium stem (12 mm × 75 mm) and a long stem (12 mm × 100 mm). In the Triathlon^®^ product line, stem diameters range in size from 9 mm to 21 mm (Stryker^®^, UK); in the present study, a non-canal filling stem diameter of 12 mm was chosen, as this represents a common size employed by surgeons for cementing.

**Fig. 1:**
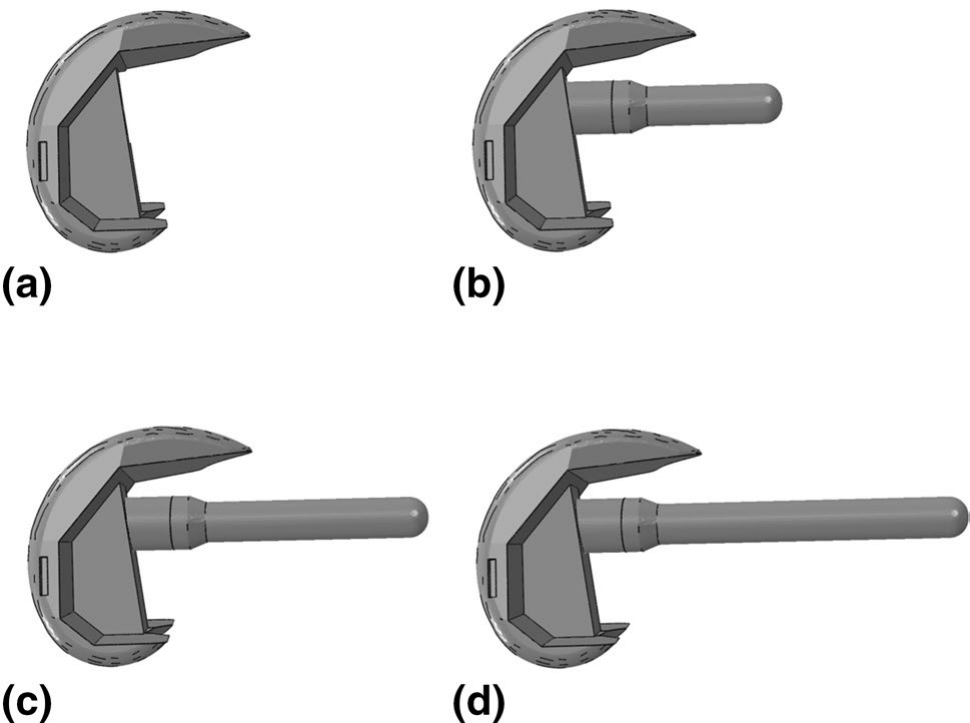
Image of all four implant types investigated in this study, **a** a posterior stabilising (PS) implant, **b** a total stabilising implant (TS) with short stem (12 mm × 50 mm), **c** a TS implant with medium stem (12 mm × 75 mm) and **d** a TS implant with long stem (12 mm × 100 mm)

The four implant configurations investigated in this study are presented in Fig. 1.

### Cement layer

**Fig. 2:**
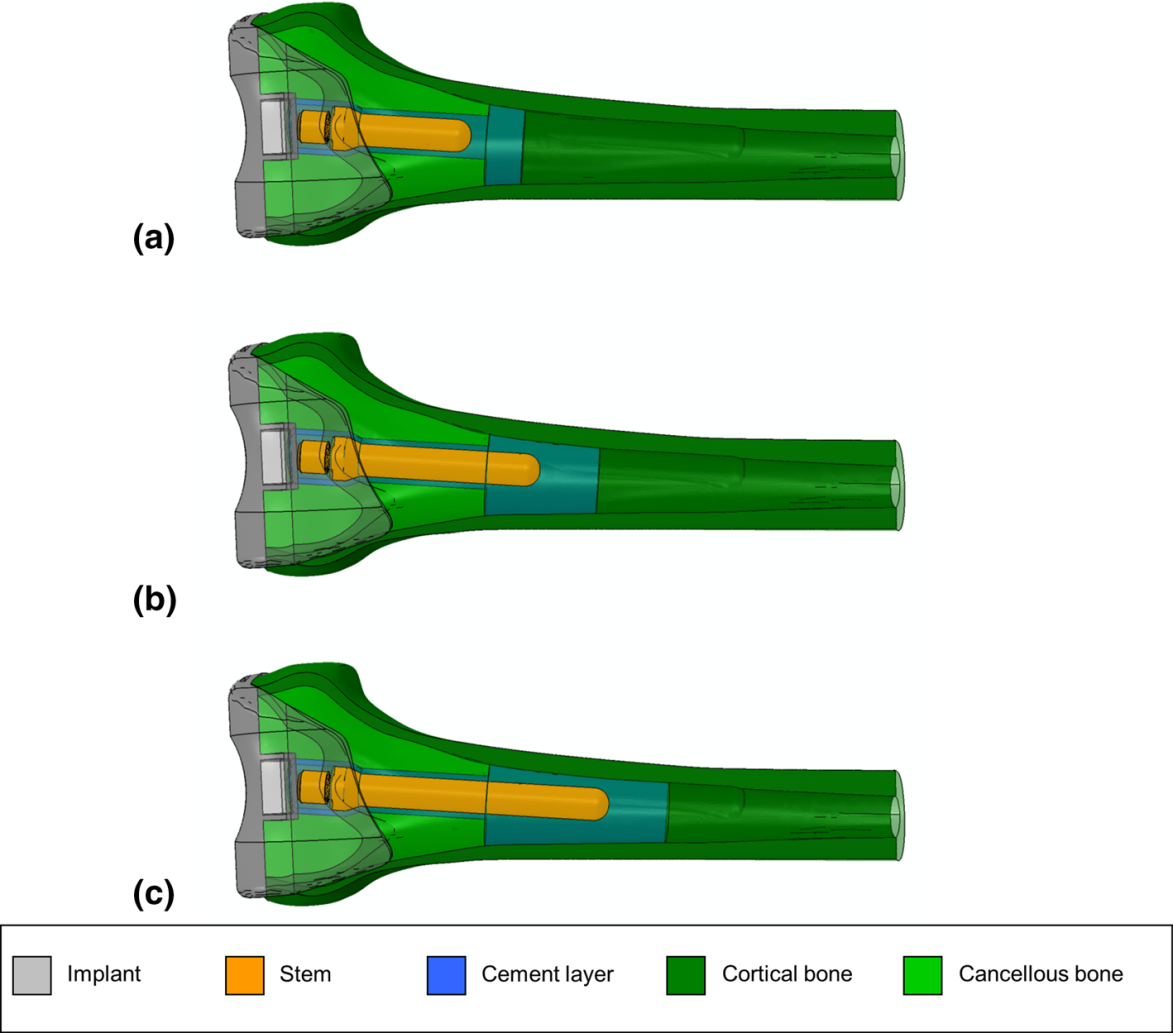
Semi-transparent rendering of **a** a femur implanted with 50-mm stem, **b** a femur implanted with 75-mm stem and **c** a femur implanted with 100-mm stem, with cement, bone and prosthesis regions indicated through the *colour coded legend at the bottom*

For simplicity, the cement layer was only modelled explicitly from the back surface of the implant box along the stem to a distance of 20 mm past the stem tip, representing where the cement is retained by the cement restrictor in the clinical setting [16]. The cement layer then extended outward to the endosteal surface of the cortical bone modelling permeation of the cement through the cancellous structure to the cortex, as is achieved in vivo through pressurisation of the cement into the bone with a cement gun. An assembled view of the cemented stemmed implants is presented in Fig. 2, with implant, stem and bone structures identified.

### Interface conditions

All interfaces were assumed to be fully bonded; the implant–stem assembly was fully tied to the internal surface of the cement, and the external surface of the cement layer tied to the surrounding bone. No relative motion was allowed between the structures modelling full cemented fixation of the implant assembly into the bone.

### Material properties

The femoral component, femoral stem, cement layer and cancellous bone regions were assumed to behave in a linear elastic, isotropic and homogeneous manner. The cortical bone structure, on the other hand, was modelled as inhomogeneous with endosteal thinning of the cortex, using the methodology introduced by Conlisk et al. [13], to simulate an old osteoporotic patient.

In brief, this method involves creating a distribution of Young’s modulus which varies linearly from endosteum to periosteum, based on previously reported variations [14], and then offsetting the location of the endosteal Young’s modulus by 50%, in line with population values [6, 28], to simulate trabecularisation of the cortical bone. The values of Young’s moduli and Poisson’s ratio applied to all structures in this study are presented in Table 1.

**Table 1:** Material properties applied to finite element model

Component	Young’s modulus *E* (N/mm^2^)	Poisson’s ratio (*ν*)
Cancellous bone	155	0.3
Cortical bone (endosteum)	7000	0.3
Cortical bone (periosteum)	16,700	0.3
Cement	2280	0.3
Femoral component (Co–Cr)	210,000	0.3
Femoral stem (ti-6al-4v)	110,000	0.3

### Loading and boundary conditions

**Fig. 3:**
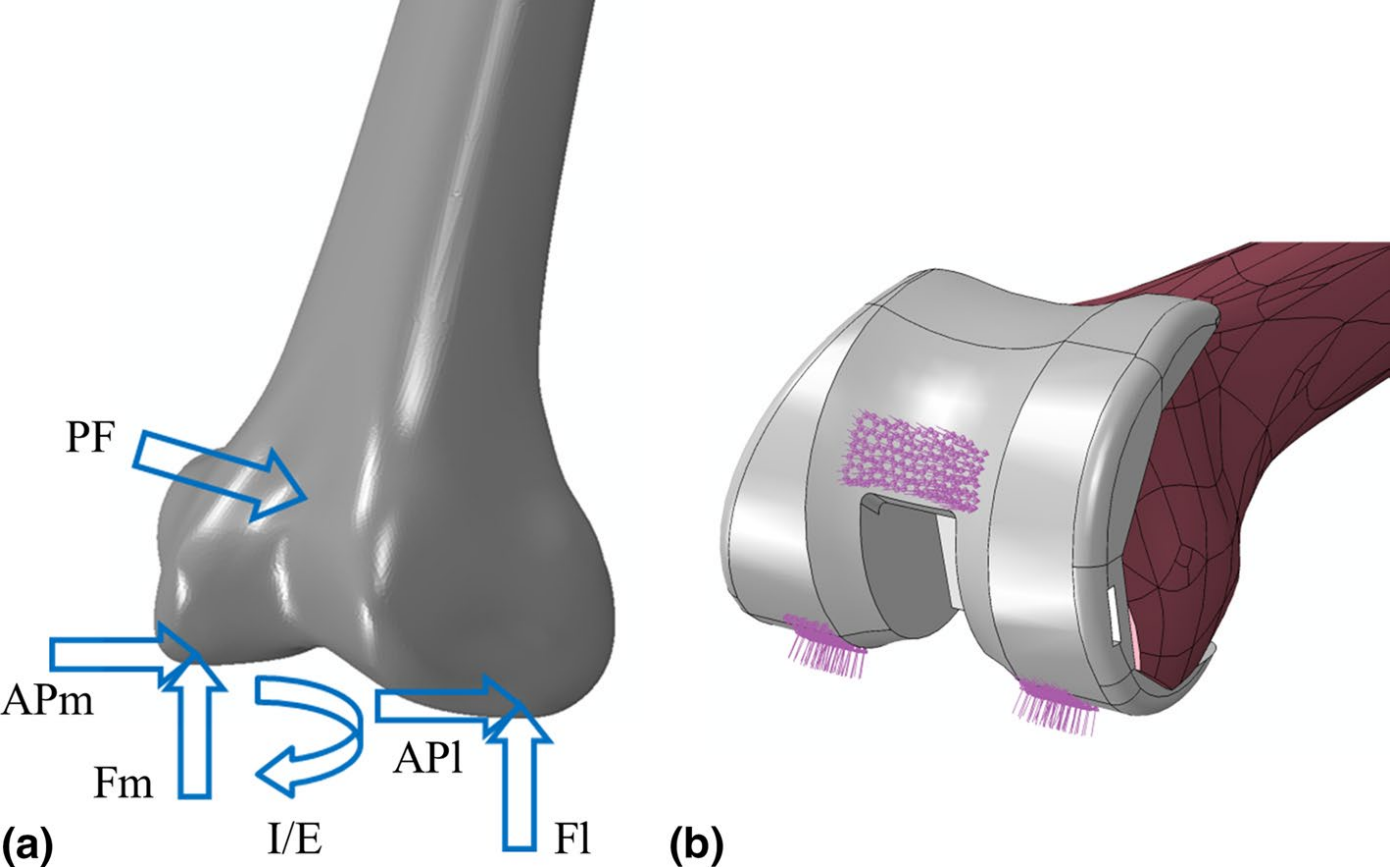
**a** Arrangement of forces at the distal femur, and **b** contact areas over which they are applied for 48° flexion

A single flexion angle (48°), representative of maximum load bearing, during the stance phase of gait for a normal walking cycle was investigated in this study and modelled as a static load step. This flexion angle was select for investigation, as previous work has shown maximum periprosthetic stresses and strains to coincide with maximum load bearing [13] for a normal walking. The loads acting on the femur at 48° flexion comprised of six separate components (Fig. 3): the patella–femoral force (PF); the medial and lateral components of the joint normal force (Fm and Fl); the medial and lateral components of the joint shear force (APm and APl); and the internal/external moment (IE). The exact magnitudes applied for each component of force are indicated in Table 2. All forces were applied as distributed pressure loads over realistic contact areas [12], with a 60–40% (medial/lateral) load distribution acting across the condyles assumed for the axial components of force [30].

**Table 2:** Forces used in the FE analyses for 48^°^ flexion

	48^°^
Medial force Fm (N)	1160
Lateral force FL (N)	773
Medial anterior–posterior force APm (N)	−3
Lateral anterior–posterior force APl (N)	−3
Patella–femoral force PF (N)	567
Internal–external moment IE (Nmm)	−7029

Values were obtained from previous in vivo telemetric implant studies [4, 35], normalised in terms of body weight and then applied to the FE model for an assumed average body weight of 775 N

Each femur model was truncated at the mid-diaphysis (approximately 242 mm from the distal surface) and all its translations/rotations fixed. This manner of fixation is consistent with many previous FE [1, 5, 10, 12, 36–38] and experimental investigations [5, 8, 11, 22].

To ensure accuracy of the numerical solution, a maximum allowable element edge length of 2 mm was applied to all models. Based on convergence studies, a further reduction in element edge length produced a negligible (2%) change in calculated displacements/stresses, while dramatically increasing simulation runtime. Final FE meshes typically comprised of >290,000 quadratic tetrahedral elements (C3D10 M). Simulation runtime for each model was in the region of 2.5 h on a dual core Intel i5 laptop with 8 GB of RAM.

### Comparative analysis

**Fig. 4:**
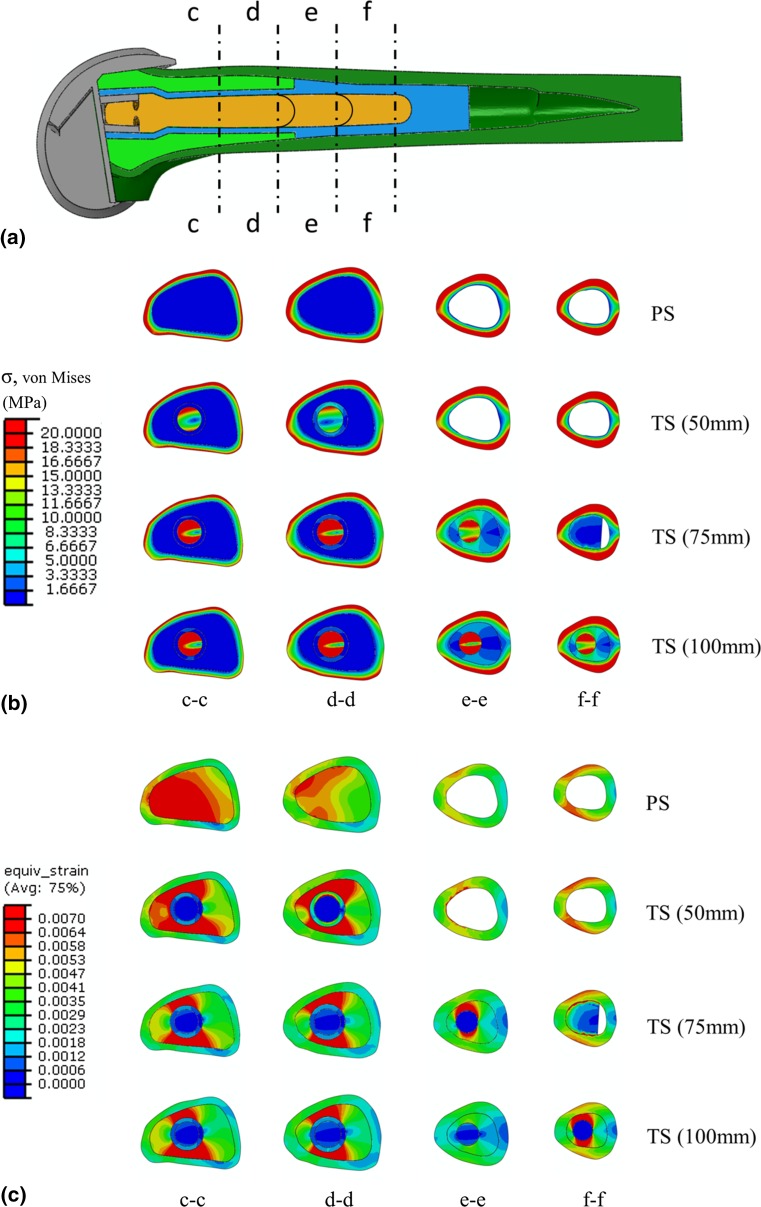
Showing **a** the location of each of the transverse sections through the femur, **b** the resulting plots of von Mises stress and **c** equivalent strain at the transverse sections for all cases investigated

A number of transverse sections through each femur (Fig. 4a) were taken to better understand the impact of cemented stems on its mechanical environment and to examine how alterations to the stresses and strains distally may affect other regions of the femur (e.g. the region surrounding the end of the stem). The section c–c represents a location just above the implant. Section d–d is taken at the location near the end of the 50-mm stem, section e–e at a location near the end of the 75-mm stem and finally section f–f at a location near the end of the 100-mm stem. It should be noted that to ensure consistency of results, all transverse sections were examined for each case.

## Results

It can be seen from Fig. 4b that the majority of the stress (von Mises) is transmitted through the stem–cement construct leading to a reduction in stress distally for femurs implanted with stemmed prostheses (section c–c). As the end of each stem is approached, section d–d for the TS implant with 50-mm stem, section e–e for the 75-mm stem and section f–f for the 100-mm stem, it can be seen that the stress in the cement increases, indicating increased load transfer at the stem–cement and cement–bone interfaces, leading to a slight increase in cortical regions above the end of the stem. Similarly in Fig. 4c, it can be observed from the plots of equivalent strain that the added stiffness of the stems serve to reduce the level of strain in the distal femur.

**Fig. 5:**
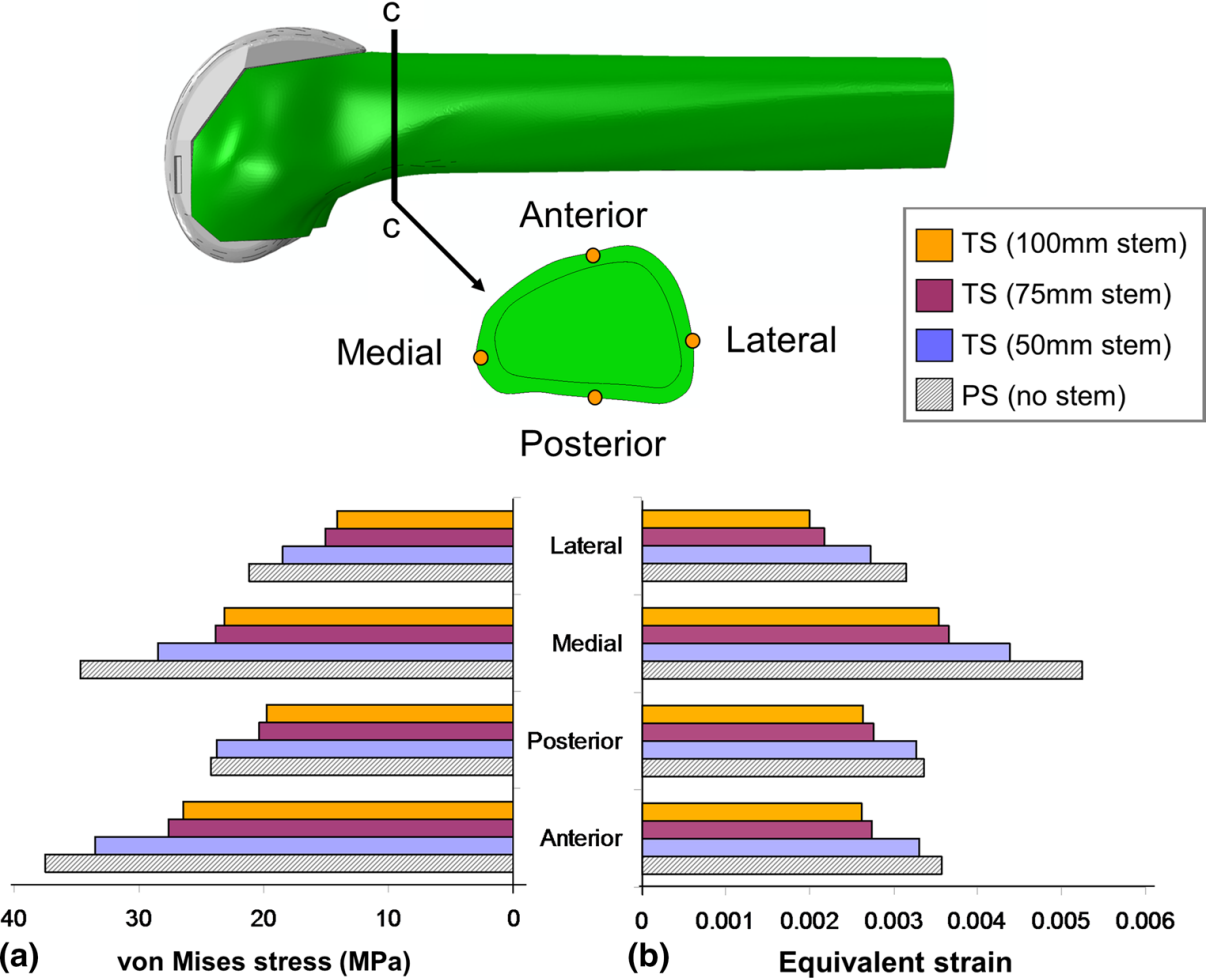
**a** von Mises stress and **b** equivalent strain at each of the points of interest for varying stem length

To characterise the influence of stemmed femoral prosthesis on the periprosthetic mechanical environment in a more quantifiable manner, the values of von Mises stress and equivalent strain were recorded at four cortical points of interest, at the location of the transverse section c–c, as shown in Fig. 5a, b, respectively. It can be seen for all stemmed femoral components that the largest values of strains occur on the medial cortex and the largest reported values of stress act on the anterior femoral cortex. The short stem (50 mm) is observed to have a response closest to that of the stemless PS implanted femur and leads to the highest levels of periprosthetic stress of all the revision stemmed implants investigated.

If we compare the average values of stress and strain from all stemmed prostheses to that of the PS implanted femur, the percentage reduction in stress and strain due to stem length can be determined (Table 3). The table shows that on average the 50-mm stem resulted in an overall decrease of approximately 11% in periprosthetic stress and 10% in periprosthetic strain, the 75-mm stem lead to a 26% reduction in both stress and strain, whereas the 100-mm stem resulted in a 29% reduction in stress and strain.

**Table 3:** Overall percentage reduction in periprosthetic stress and strain for femurs implanted with stemmed implants relative to a PS implanted femur

Model	Stem length (mm)	Decrease in von Mises stress (%)	Decrease in equivalent strain (%)
PS	N/A	0.0	0.0
TS short	50	10.9	10.0
TS medium	75	25.7	25.5
TS long	100	28.7	29.3

Where for each femur, values of stress and strain are calculated based on the average of the four cortical points of interest

## Discussion

The most important finding of this study was that cemented non-diaphyseal engaging stems were beneficial in reducing periprosthetic stress. The level of reduction was observed to be related to the length of the stem employed, with a medium-length stem representing the best balance between bone preservation at the time of surgery and reduction in periprosthetic stress following implantation.

Prosthesis-induced stress shielding is frequently observed following TKA [1, 25, 31, 33, 36, 37]. However, it is important to recognise that internal stresses need to be in equilibrium with externally applied forces; as a result, a reduction in stress in some regions will lead to an increase in stresses in others [31]. In the context of the current study, the observed reduction distally leads to an increase in stress proximally in the cement and stem structures. However, it should be noted that as the load transfer is distributed along the length of the stem–cement construct, the overall result is that implanted femurs with longer stems (i.e. 75 and 100 mm stems) are subject to slightly lower stresses and strains proximally than the stemless or short-stemmed cases. Furthermore, as the cortex is significantly thicker in the mid-shaft, it may be less susceptible to fracture than the much thinner and less stiff cortical bone structure found immediately adjacent to the implant.

Stems are widely accepted as being necessary to ensure successful outcomes during revision TKA, particularly in instances of severe bone loss [15, 24], where bone grafts are required to replace damaged or diseased host bone. In this capacity stems serve to transmit the load to healthy bone structures above the implant and away from the graft, thereby allowing it to fully incorporate into the host structure without risk of damage and overloading [15]. Stemmed prostheses have also found use in the treatment of ligament laxity through aiding in the stability of more constrained implant types [18]. It has also been suggested previously that the addition of a stem may also serve to decrease the likelihood of periprosthetic femoral fracture following notching of the anterior femoral cortex [7, 19].

In the present study, the novel application of stems as a preventative measure against periprosthetic fracture in older patients with osteoporosis was investigated. It was found that the addition of a cemented stem to the distal femur caused a reduction in periprosthetic stress. The short cemented stem (50 mm) caused a reduction of approximately 11%, while longer stems (75 and 100 mm) were seen to result in reductions of 26–29% when compared with a stemless PS implanted femur. The reduction in strain was observed to follow a similar trend with respect to stem length. Moreover, the reduction in strain which occurred as a result of the medium stem being found to be of a similar magnitude to that found previously in a study of fracture risk following notching [7]. Based on the findings of the present study, it can be seen that the stem length is an important parameter for the reduction in periprosthetic stress in older osteoporotic patients. Of the three stems investigated, the use of a 75 mm cemented stem is recommended, as this stem affords a reduction in stress almost comparable to the 100-mm stem (e.g. less than 4% difference between the two) while also preserving a greater level of bone stock (e.g. less bone removed during implantation of smaller stems). It is also known that shorter stems in general are easier to fit at the time of operation and are less influenced by the curvature of the femur [24].

Moreover, the selection of a smaller stem has been suggested on the grounds of preserving bone density in the femur post-implantation. In a study by van Lenthe et al. [38] on bone remodelling around revision prosthesis, the influence of two stemmed femoral components (thick and thin) relative to a primary implant was investigated. The findings of van Lenthe et al. suggested that a femoral component with a thin stem performed in a similar manner to the primary stemless implant and had a much more favourable rate of bone loss than a femoral component with a thick stem.

It must be noted that while stems provide many immediate benefits, several clinical reports also suggest a higher incidence of fracture in stemmed versus stemless prosthesis. Meek et al. [21] reported a fracture risk of 0.6% for primary and 1.7% for revision knee arthroplasty at 5 years; these values increased to 1.3 and 2.2%, respectively, at 10 years. Meek and colleagues identified female patients over 70 years of age to be most at risk of fracture following knee replacement. A study by Singh et al. [32] spanning a 19-year period reported similar periprosthetic fracture rates following primary (1.1%) and revision (2.5%) knee arthroplasty. Interestingly, very different conclusions were drawn with respect to at-risk patient groups by these two studies. The somewhat contradictory evidence surrounding periprosthetic femoral fracture following primary and revision TKA indicate that the exact mechanisms at work are quite complex and may warrant closer examination. However, it is important to recognise that the implantation of a revision femoral prosthesis in the setting of primary TKA is not the same as implantation of the same prosthesis during revision surgery. Both scenarios will have very different initial conditions, particularly with respect to bone quality for fixation of the prosthesis. It must therefore be considered that the clinical observances that revision stemmed prostheses have a higher fracture rate than primary stemless implants, may not be directly applicable to the present study, as a wide array of factors may influence the outcome in revision surgery (e.g. age, gender, and health). Indeed, a recent retrospective study by Barlow et al. [2] highlighted comparable short-term outcomes (at 49 months) for stemmed and conventional TKA when used in a primary setting.

The present study has some limitations. In this study, only a single diameter of stem was considered, with stem length varied using three common sizes. However, most manufacturers provide a range of different stem diameters, sizes, lengths and end designs, as such alterations to any one of these parameters may also influence the load sharing at the bone–prosthesis interface. It is therefore recommended that further studies be conducted to assess the importance of other stem parameters. Another consideration is that only cemented stems were investigated. Cementing in all cases was modelled on a fully bonded metaphysis and pressurising of cement along the stem out to the cortex to achieve optimum load transfer, which may not be fully representative of in vivo conditions. If full contact was not achieved, then this could severely reduce the load sharing capacity of the construct [38] and limit its effectiveness for preventing periprosthetic fracture. Furthermore, a reduction in load sharing may lead to bone loss, and eventual loosening due to overloading of the surrounding cancellous bone [34]. Should loosening occur distally, the accompanied reduction in load sharing could result in the majority of load being transmitted solely through the femoral component–stem assembly, increasing the likelihood of stem junction failure and femoral fracture occurring [12, 17, 20, 23]. Periprosthetic stresses resulting from maximum load bearing during walking were examined in this study. Other patterns of gait (e.g. ascending/descending stairs, and squatting) may result in very different distributions of stress in the femur. However, due to the comparative nature of this study, it is unlikely that the conclusions drawn would be significantly affected by alterations to the pattern of loading (once applied uniformly across all model variations). This study examined the initial response of the femur immediately following implantation. It is recognised that bone is dynamic and will remodel in response to the presence of an implant. Therefore, if predicting the long-term survival of the prosthesis is of interest (e.g. loosening or fracture), incorporation of a complex bone remodelling framework, e.g. [27], would be required to adequately capture the response of the femur to disease progression. Finally, only a single femoral geometry with osteoporotic bone properties was considered in this study; future studies should examine whether the observed benefits of cemented stems extend to other femoral geometries and patient types.

Despite these limitations, the findings of this study may aid Surgeons to achieve better post-implantation outcomes for older osteoporotic patients, through an enhanced understanding of how different implant types affect the underlying mechanics of the bone.

## Conclusion

The findings of the present study suggest that a small-diameter medium-length cemented stem could have beneficial applications in reducing periprosthetic stress and therefore fracture risk in elderly patients with osteoporosis. It is recommended that clinical studies should be conducted to confirm the findings of this biomechanical modelling study. The outcomes of which, in conjunction with the work presented here, could then help to better inform surgical treatment of patients in this particular group.
